# Attack rate, case fatality rate and determinants of measles infection during a measles outbreak in Ethiopia: systematic review and meta-analysis

**DOI:** 10.1186/s12879-023-08757-0

**Published:** 2023-11-02

**Authors:** Mengistie Kassahun Tariku, Daniel Tarekegn Worede, Abebe Habtamu Belete, Simachew Animen Bante, Sewnet Wongiel Misikir

**Affiliations:** 1 Department of Public Health, College of Health Science, Debre Markos University, Debre Markos, 269 Ethiopia; 2 Department of Midwifery College of Health Sciences, Bahir Dar University, Bahir Dar, 79 Ethiopia; 3 Department of Laboratory Technology, Felege Hiwot Comprehensive Specialized Hospital, Bahir Dar, 680 Ethiopia

**Keywords:** Measles, Outbreak, Review, Ethiopia

## Abstract

**Background:**

Although Ethiopia is working towards measles elimination, a recurrent measles outbreak has occurred. To take appropriate measures, previously, many fragmented and inconsistent outbreak investigations were done, but there is no consolidated evidence on attack rate, case fatality rate, and determinants of measles infection during the measles outbreak. This systematic review and meta-analysis aimed to identify cumulative evidence on attack rate, case fatality rate, and determinants of measles infection during the outbreak.

**Methods:**

A systematic literature review and Meta-analysis was used. We searched Google Scholar, Medline/PubMed, Cochrane/Wiley Library, EMBASE, Science Direct, and African Journals Online databases using different terms. Investigations that applied any study design, data collection- and analysis methods related to the measles outbreak investigation were included. Data were extracted in an Excel spreadsheet and imported into STATA version 17 software for meta-analysis. The I^2^ statistics were used to test heterogeneity, and ‘Begg’s and ‘Egger’s tests were used to assess publication bias. The odds ratio (OR) with a 95% confidence interval (CI) was presented using forest plots.

**Results:**

Eight measles outbreak investigations with 3004 measles cases and 33 deaths were included in this study. The pooled attack rate (A.R.) and case fatality rate were 34.51/10,000 [95% CI; 21.33–47.70/10,000] population and 2.21% [95% CI; 0.07-2.08%], respectively. Subgroup analysis revealed the highest attack rate of outbreaks in the Oromia region (63.05 per 10,000 population) and the lowest in the Amhara region (17.77 per 10,000 population). Associated factors with the measles outbreak were being unvaccinated (OR = 5.96; 95% CI: 3.28–10.82) and contact history (OR = 3.90; 95% CI: 2.47–6.15).

**Conclusion:**

Our analysis revealed compelling evidence within the outbreak descriptions, highlighting elevated attack and case fatality rates. Measles infection was notably linked to being unvaccinated and having a contact history. Strengthening routine vaccination practices and enhancing contact tracing measures are vital strategies moving forward.

## Introduction

A measles outbreak investigation is the diagnosis and confirmation of a suspected measles outbreak through the urgent and intelligent use of appropriate procedures to take suitable measures toward controlling the outbreak [1, 2]. The purposes of measles outbreak investigation are to assess the extent of the outbreak, search for additional cases, identify the source of the epidemic and the population at risk, and institute timely case management to reduce morbidity and mortality and prevent future attacks [1–3].

Measles is the most contagious viral human disease, commonly resulting in wide-ranging outbreaks [4, 5]. Any person with fever, maculopapular rash, cough, coryza/runny nose, or conjunctivitis/ red eyes is a suspect of a measles case. When the suspect measles case is positive for an immunoglobulin M (IgM) antibody test, it is called a confirmed measles case [3, 4]. Measles is transmitted through droplets from nose, mouth, or throat secretions [6]. It has a secondary attack rate of 90% in susceptible household and institution contacts [4, 5]. The most severe complications of measles are blindness, pneumonia, and encephalitis, leading to case fatality rates ranging from 0.1% in industrial countries to 15% in developing countries [7, 8]. Severe complications of measles are more common in children under 5 years and adults older than 20 years old [8].

A suspected measles outbreak is the occurrence of five or more reported suspected measles cases in one month per 100,000 population living in a geographic area such as a kebele, woreda, or health facility catchment area. A confirmed measles outbreak is the occurrence of three or more laboratory-confirmed suspected measles cases in one month per 100,000 population living in a geographic area such as a kebele, woreda, or health facility catchment area [9].

An outbreak occurs when the accumulated number of susceptible individuals is greater than the critical number of susceptible individuals for a given population to sustain transmission [2, 3]. Approximately 15% of children vaccinated at nine months of age and 5% of those vaccinated at 12 months of age fail to seroconvert and are thus not protected after vaccination [3].

Even though there is a significant success in global measles mortality reduction and elimination [7], more than 100,000 measles deaths occurred in 2017 [10]. More than 95% of these deaths occurred in low-income countries [11]. Although the African region and Ethiopia are working towards measles elimination by 2020 [3], Ethiopia is still the 4th top country in the world in the burden of measles cases [1].

An outbreak investigation is conducted within 3 h of notification of a suspected measles outbreak [12]. Timely outbreak investigation and response may reduce morbidity, mortality, and the spread of the outbreak [3]. It is also one of the most important measures for eliminating measles [13].

In 2019, Ethiopia introduced the measles vaccine second dose (MCV2) vaccination into the routine immunization program in the second year of life for measles elimination [10]. Despite these efforts, in Ethiopia, a recurrent measles outbreak has occurred [14].

Ethiopia initiated a field epidemiology and laboratory training program in 2009 [15]. This program has played a significant role in improving outbreak detection and investigation. There are many single, fragmented measles outbreak investigations in Ethiopia, but there is a lack of collective evidence on the attack rate, case fatality rate, and determinants of measles infection during the outbreak. Therefore, this systematic review and meta-analysis aimed to measure the attack and case fatality rates and identify measles infection determinants during the outbreak investigation.

## Methods

### Study design and searching methods

This investigation used a systemic review and Meta-analysis of published measles outbreak investigations. Published investigations were searched in Google Scholar, MEDLINE/PubMed, Cochrane/Wiley Library, EMBASE, Science Direct, and African Journals Online databases. The search terms used were “Measles attack rate OR Measles incidence OR Measles case fatality rate OR Measles outbreak investigation OR Measles outbreak ORMeasles epidemic OR Determinant of measles infection OR investigation of measles outbreak or Risk factors of a measles outbreak and Ethiopia.“

### Investigation selection and eligibility criteria

This systematic review and meta-analysis encompassed all published studies that examined measles outbreaks in Ethiopia. The study design of the included investigations was not restricted. The review incorporated measles outbreak studies published in English between 2010 and 2022.

### Outcomes

This review has outcome variables for the measles attack rate, which is calculated as the number of people who became ill from measles divided by the number of people at risk for the measles disease, and the case fatality rate, which is calculated as the number of measles-related deaths by the number of measles total cases [16]. The determinants of contracting a measles virus were also outcome variables. Vaccination status and contact were determinants of measles virus infection.

### Quality assessment

The included investigations were assessed for quality, with only high-quality investigations included in the meta-analysis. Two independent authors assessed each investigation’s quality by using an adapted Newcastle- Characteristics of included investigations tool [17]. Generally, the tool consists of three sections; the first section is ranked out of five stars and reflects the methodological quality of each primary investigation. The second section compares the study outcomes or exposures with the possibility of adding two stars. The third section focuses on each primary study’s outcome and statistical analysis, with the possibility of three stars [18]. We compared the quality assessment scores of the investigations and determined any difference before computing the final assessment score. Investigations with a score of > 7 out of 10 scales were assumed to be high quality, revealing that all investigations were eligible.

### Data extraction and management

Before data extraction, a standard tool was adapted by reviewing different kinds of literature. The data extraction tool included such information as Author name, publication year, journal name, publication year, study design, study period, study setting, sample size, descriptive data analysis (attack rate, case fatality rate, outbreak duration, and vaccination status), and factors of a measles outbreak. The data extraction tool was developed independently by two authors and revised by another two authors. Before data collection, all authors agreed on the tool.

### Data analysis

Data was collected and organized in an Excel spreadsheet and transferred to STATA version 17 software to conduct the meta-analysis. The primary focus of the systematic review and meta-analysis was on the comprehensive assessment and consolidation of attack rates, case fatality rates, and factors influencing measles infection.

The combined attack and case fatality rates and their corresponding 95% confidence intervals (CI) were calculated by employing each study’s attack rate, case fatality rate, and standard error information. The results were visually represented through forest plots, illustrating the 95% CI for attack rates, case fatality rates, and odds ratios (OR) related to factors associated with measles virus infection.

Subgroup analysis was conducted to investigate potential disparities, considering factors such as region and study period. A random effects model was utilized in the meta-analysis to account for heterogeneity across the included studies [19].

### Heterogeneity and publication bias

The investigation’s heterogeneity was tested using the I squared (I^2^) test statistic and its corresponding p-value [20]. A p-value less than 0.05 was used to declare heterogeneity [21]. I^2^ 25, 50, and 75% statistics were used to declare low, moderate, and high heterogeneity, respectively [19]. ‘Egger’s and ‘Begg’s tests are commonly used to assess potential publication bias in a meta-analysis, and a p-value less than 0.005 to state its significance [22, 23].

## Results

### Study selection

**Fig. 1:**
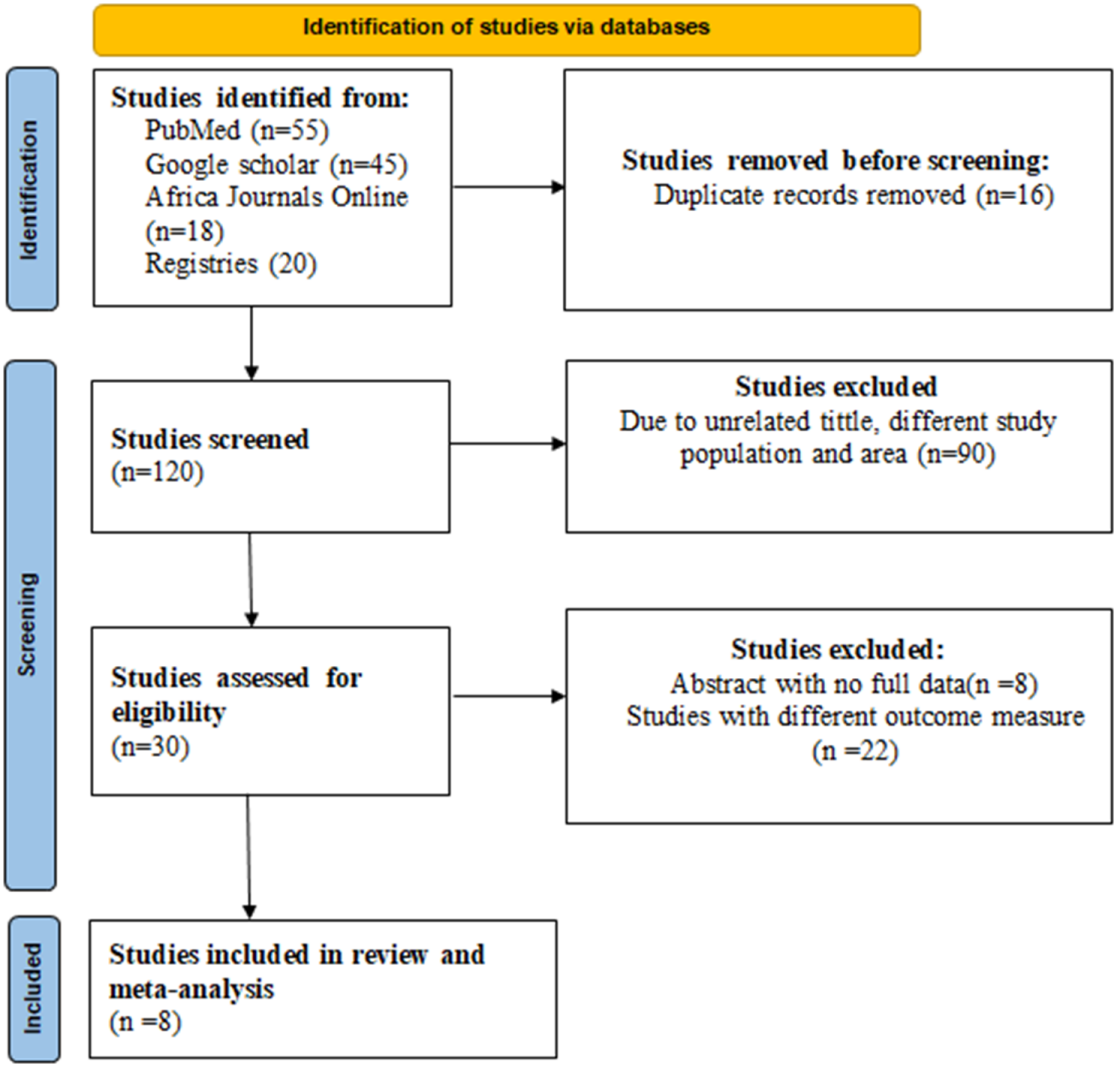
Flow Diagram of measles outbreak investigation included in systematic review and meta-analysis in Ethiopia, 2022

Through electronic database searches, we accessed a total of 136 records. Initial screening was done by assessing titles, abstracts, and complete article reviews, excluding 90 articles based on title and abstract evaluations. Subsequently, 30 articles underwent eligibility evaluation, with 22 being excluded due to incomplete reporting. Ultimately, 8 investigations met the criteria and were incorporated into this meta-analysis (Fig. 1).

### Characteristics of included investigations

This systematic review and meta-analysis encompassed eight distinct investigations of measles outbreaks, involving a cumulative count of 3004 confirmed measles cases and resulting in 33 reported fatalities (age range: 1 month to 75 years) [17, 24–30]. More than three-fifths (62.5%) of the investigations were case-control study designs [24, 27–30], and three articles were descriptive cross-sectional study designs [17, 25, 26]. All included outbreak investigations used both primary and secondary data sources, which were investigated from 2009 to 2021. Four articles were conducted in the Amhara region [24, 25, 28, 29], three in the Oromia region [17, 27, 30], and one in the Somalia region [26] (Table 1).

**Table 1:** Summary Characteristics of studies included in a systematic review and Meta-analysis of a measles outbreak investigation in Ethiopia, 2022

Authors Name	Study setting/region	Study design	Study period	Sample size	Duration of the outbreak in days	AR per 10,000 population	CFR (%)
Yusuf M. et al., 2017	Somali	Cross-sectional	February to March 2016	406	70	28.2	1.2
Tesfaye, A., et al., 2019	Amhara	Case-control	February 2017	261	74	12.4	0
Girmay. A., et al., 2019	Amhara	Case-control	May 18 to 30, 2016	87	21	69.9	0
Tsegaye, G., et al., 2022	Oromia	Case-control	March 25 to April 9/2021	218	48	120	7.15
Belda, K., et al., 2017	Oromia	Cross-sectional		1059	86	8.1	0.2
Kalil, F.S., et al., 2020	Oromia	Case-control	March 18 to April 29, 2019	1155	117	63	0.5
Tariku, M.K. et al. 2019	Amhara	Case-control	July 13-Agust 1/2018	190	18	1.18	2.6
Aragaw, M. et al. 2012	Amhara	cross-sectional	May 30-July 30/2009	97	32	4.1	13.4

### Attack rate

**Fig. 2:**
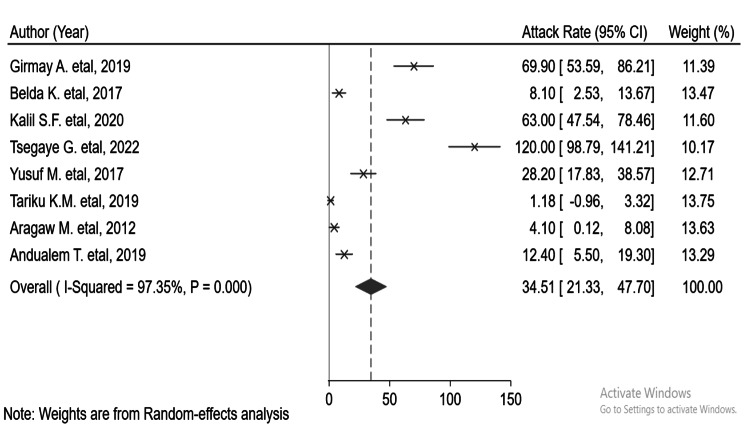
Pooled attack rate of measles during an outbreak in Ethiopia. p-value = 0.00047 and ‘Begg’s = 0.00053 (Fig. 2)

This systemic review and meta-analysis’s pooled attack rate (A.R.) was 34. 51 (95% CI: 21.33–47.70) per 10,000 population [17, 24–30] (Fig. 2). Higher and more significant heterogeneity ( I^2^ = 97.35% and p-value < 0.000). Considerable publication bias was also detected, with Eggers, .

### Case fatality rate (CFR)

**Fig. 3:**
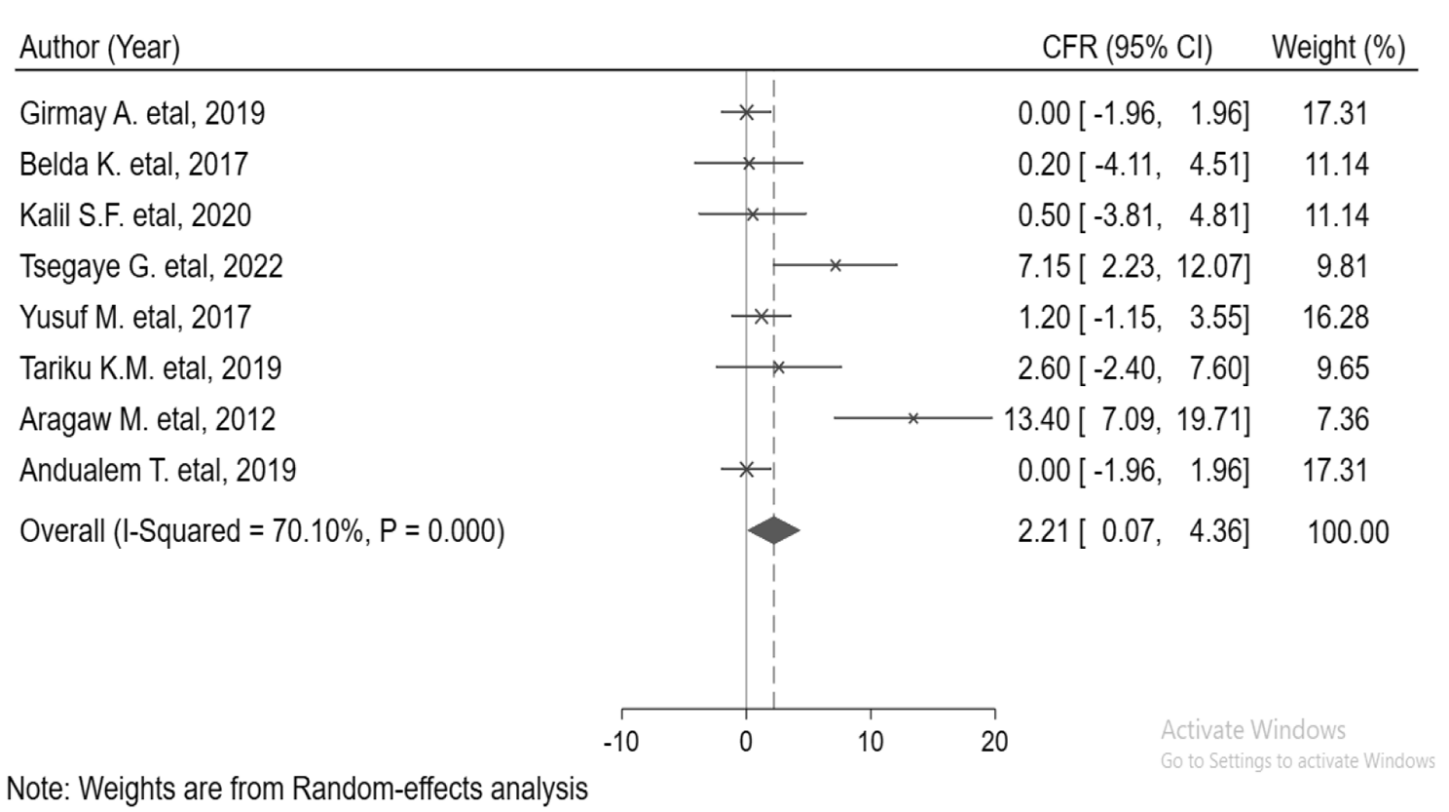
Pooled case fatality rate during the measles outbreak in Ethiopia

The overall pooled CFR of this investigation was 2.21% (95% CI: 0.07-4.36%) [17, 25–28, 30]. There is moderate and significant heterogeneity, I^2^ = 70%, and a p-value of < 0.00001. Significant publication bias was also detected, Begg’s = 0.00853 and ‘Egger’s = 0.00436 (Fig. 3).

### Subgroup analysis

Subgroup analysis of attack rate done by region showed that the highest attack rate was observed in the Oromia region, 63.05 (95% CI; 47.54–126.12) per 10,000, and the lowest in the Amhara region, 17.77 (95% CI; 5.58–29.95) per 10,000 population. A higher attack rate of measles was observed after 2019, 91.02 (95% CI; 35.17 -146.87) per 10,000 population (Table 2).

**Table 2:** Subgroup analysis for an attack rate of measles outbreak in Ethiopia

Subgroup	Number of Studies	Total Sample size	Attack Rate Per 10,000 (95% CI)	Heterogeneity statistics		
By Region				Tau-Squared	I^2^	P-value
Amhara	4	635	17.77(5.58–29.95)	137.00	96.0	0.00001
Oromia	3	2394	63.05(47.54-126.12)	304.47	98.0	0.00001
Somalia	1	406	28.20(17.83–38.57)			
By Study Period						
Before 2019	6	2100	17.45(7.94–26.96)	123.96	95.0	0.00001
After 2019	2	1335	91.02(35.17-146.87)	153.48	94.0	0.00001

In subgroup analysis, the Amhara region, 2.83% (95% CI; 0.86-6.52%), and the Oromia region, 2.46% (95% CI; 1.77 -6.70%), had almost equal CFR. A higher case fatality rate was reported after 2019, at 3.35% (95% CI; 3.10-9.81%) (Table 3).

**Table 3:** Subgroup analysis for a case fatality rate of measles outbreak in Ethiopia

Subgroup	Number of Studies	Total Sample size	Case Fatality Rate	Heterogeneity statistics		
By Region				Tau-Squared	I^2^	P-value
Amhara	2	287	2.83(0.86–6.52)	10.46	82.44%	0.001
Oromia	3	2394	2.46(1.77–6.70)	8.75	62.35%	0.070
Somalia	1	406	1.2(1.15–3.55)			
By Study Period						
Before 2019	4	1752	1.38(0.01–2.77)	1.08	89	0.000
After 2019	2	1335	3.35(3.10–9.81)	18.94	86	0.008

According to the existing evidence, vaccination status and contact history were the main determinants of measles infection.

### Vaccination status

**Fig. 4:**
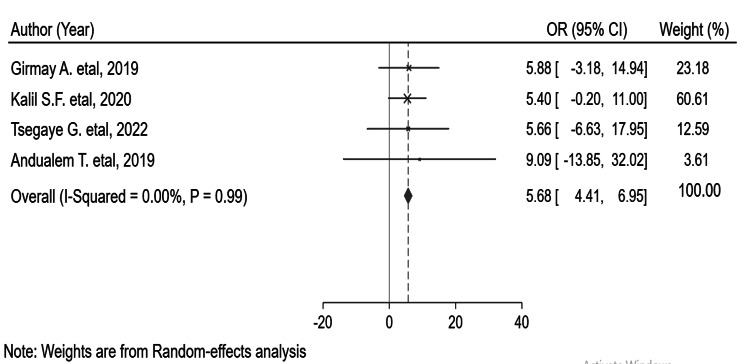
Pooled odds ratio between vaccination status and contracting the measles virus

The vaccination status measured as being vaccinated or unvaccinated strongly influenced contracting the measles virus in four outbreak investigations [24, 27, 29, 30]. The meta-analysis revealed that individuals who had not been vaccinated against the measles virus were six times more likely to contract the virus (OR = 5.68; 95% CI: 4.41–6.95). The heterogeneity test showed that there is no evidence of heterogeneity (I^2^ = 0, and p-value = 0.99). However, there is a significant publication bias (‘Begg’s test = 0.00032 and ‘Egger’s test = 0.0043) (Fig. 4).

### Contact history

**Fig. 5:**
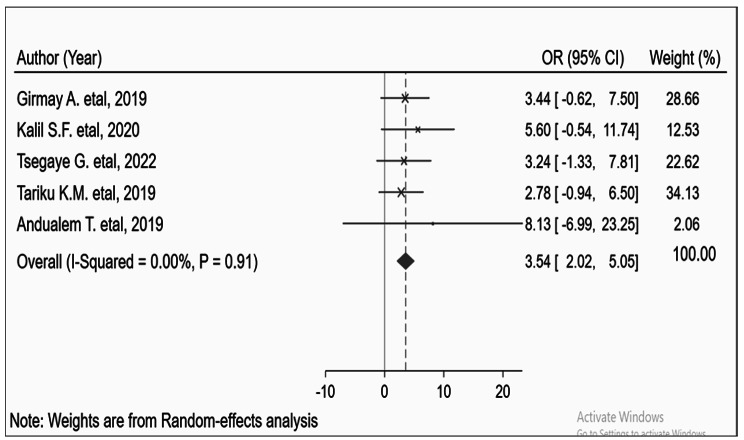
Pooled odds ratio between contact history and contracting the measles virus

A contact history significantly affected the acquisition of the measles virus [24, 27–30]. It was identified that individuals who had a contact history with measles cases 7–21 days before developing the current infection had four times (AOR = 3.54; 95% CI: 2.02–5.05) higher risk of acquiring measles infection as compared to those individuals who had no contact history during the same period. There was a significant publication bias (‘Begg’s test = 0.0036 and ‘Egger’s test = 0.0023). However, there was no evidence of heterogeneity, I^2^ = 0, and p-value < 0.91 (Fig. 5).

## Discussion

This systematic review and meta-analysis was conducted to measure the attack and case fatality rates and identify factors associated with measles infection in Ethiopia using available published articles. The overall pooled attack rate of measles during a measles outbreak was 34.51 (95% CI: 21.33–47.70) per 10,000 population. The highest and lowest attack rates were 120 per 10,000 population [30] and 1.18 per 10,000 population [28], respectively. The variation in attack rate from outbreak to outbreak could be attributed to differences in the range of the outbreak, susceptible accumulation, and nutritional status of the population. In addition to this, there might be differences in the time of outbreak investigation and response-to-action threshold. Timely outbreak investigation and response reduce morbidity, mortality, and the spread of the outbreak [3]. In three measles outbreak investigations, the highest age-specific attack rate was reported in the age group of < 1-year-old children, which was 1.7/10,000 [17], 47.6/10,000 [29], and 310/10,000 populations [30] Immunity of children can be lost in just over 2.5 months after birth or the time breastfeeding is discontinued [31]. In two investigations, the highest age-specific attack rate was reported among the age group of 15–44 years (93.8 per 10,000) [24], and under 5 years (38/1000 population) [30]. In another investigation, the highest age-specific attack rate (291 per 10,000 population) was observed at the age of 5–14 years [28]. Variations in age-specific attack rates could be attributed to differences in the immune status. A more susceptible population could be found in these age groups.

In seven measles outbreak investigations, a similar sex-specific attack rate was reported [17, 24–27, 29, 30]. Whereas in one measles outbreak investigation, a higher sex-specific attack rate (139.4/10,000) was reported among females [28]. This might be due to the fact that women are always caregivers in some parts of Ethiopia. Therefore, there could be contact with sick children 1–4 years old.

The epidemic curve was used in all included articles to describe the outbreak in terms of time [17, 24–30]. Five articles were conducted from February to April [17, 26, 27, 29, 30]. This evidence was similar to the study conducted in Nigeria [32]. This might be due to high population movement and many traditional ceremonies (weddings, religious festivals) during this season or the dryness of the season, which may make the condition favorable for measles virus transmission. Two different outbreak investigations were conducted from May to July [25] and July to August [28]. In one outbreak investigation [17], the measles outbreak persisted for 117 days. A higher attack rate (91.02/10,000) was observed in subgroup analysis after 2019. This might be due to COVID-19, which causes hesitancy in outbreak investigation and response.

In seven outbreak investigations, a place-specific attack rate was used, but a map was not utilized to identify the source of the outbreak [17, 25–30]. Whereas in one measles outbreak investigation, measles cases were not described in terms of place [24]. There was a large geographical variation in the attack rate. The observed attack rates in the Amhara region were between 1.18/10,000 population and 69.9/10,000 [24, 25, 28, 29]. In three studies in the Oromia region, the observed attack rates ranged from 8.1/10,000 to 120/10,000 [17, 27, 30]. The reported attack rate in the Somalia region was 28.2/10,000 [26]. In subgroup analysis, Oromia had the highest pooled attack rate (63.05 per 10,000) [17, 27, 30], while the lowest pooled attack rate was observed in the Amhara region (17.77 per 10,000) [24, 25, 28, 29]. This discrepancy might be the difference in clusters of non-immune individuals or due to the difference in outbreak duration. In the Oromia region, the outbreak was continuing for 48 days [30], 86 days [17], and 117 days [27] whereas, in the Amhara region, the outbreak was lasting for 18 days [28], 21 days [24], 32 days [25] and 74 days [29].

The overall pooled CFR of this meta-analysis was 2.21% (95% CI: 0.07-4.36%). In two articles, the highest age-specific CFR was reported at the age of under one year, which was 3.1% [24] and 33.3% [23]. This might be due to the immaturity of the immune system.

A higher case fatality was reported after 2019, 3.35% (95% CI; 3.10-9.81%). The variation of CFR could be imposed by the difference in case management and the community’s immunity level.

Measles vaccination provides lifelong immunity [33, 34]. Low vaccination and immunization coverage lead to an increase in the incidence of measles cases [35]. Individuals who had not been vaccinated for measles were almost six times more likely to contract the measles virus than those who had been vaccinated for measles. This is supported by the study conducted in Ohio [36]. All measles cases were unvaccinated in one measles outbreak investigation [28]. In another investigation, all measles cases had an unknown vaccination status [25]. The investigation conducted in Basoliben woreda of East Gojjam Zone, Amhara Region [29], revealed that 20% of measles cases were unvaccinated. In the above investigation, 76.7% of measles cases were vaccinated. This might be due to the problems of cold chain management and vaccine administration. On the other hand, percentage of unvaccinated measles cases by other regions was: Guji zone in the Oromia region, 75% [17], Guradamole Woreda of Bale Zone, Oromia 75.5% [30], Ginnir district of Bale zone, Oromia region, 79% [27], Sekota Zuria district, Amhara, Region, 83% [24] and Somali Region, 86.2%. This non-uniform vaccination coverage might be due to problems with accessing vaccination services or vaccine hesitancy. In six measles outbreak investigations, measles vaccine effectiveness (MVE) was not computed [24–27, 29, 30]. In two investigations, the computed MVE was 82.6% [28] and 90% [31].

The measles virus is highly contagious, and 9 out of 10 susceptible people of all ages might have contracted the virus [37]. People with contact with measles cases were four times more likely to contract the measles virus than those without no contact history. This is congruent with the study conducted in Japan, Taiwan, and China [38].

## Conclusion

Our findings revealed notable pooled attack and case fatality rates, with considerable divergence across different study regions. Measles infection was significantly linked to being unvaccinated and having a contact history. Strengthening routine vaccination strategies and intensifying contact tracing efforts emerge as imperative measures.

## Data Availability

The data sets generated during the current study are available from the corresponding author upon reasonable request.

## Abbreviations

AOR: Adjusted odd ratio
AR: Attack rate
CFR: Case fatality rate
CI: Confidence interval
MVE: Measles vaccine effectiveness
